# The War on the Dental Colleges

**Published:** 1896-02

**Authors:** 


### Editorial.

### THE WAR ON THE DENTAL COLLEGES.

When the attack on dental colleges was made in the American Dental Association at Asbury Park, it was not generally considered a remarkable or even a novel outbreak of illogical utterances, but it has evidently stirred up the college mind as never before, and we have had line upon line given us to read in which there is intermingled much truth and error. The question may be asked, "Why were not some of these things said at the meeting?" There was but one reply made to the charges when the entire body of college men present should have claimed the floor, and demonstrated by force of numbers and weight of intellect that they repudiated the defamatory remarks. Silence seemed to give an assent to these, and it was subsequently openly stated that the colleges could not and dared not deny the imputations cast upon their work.

From present indications we are on the eve of a sharply-contested war between collegemen and non-collegemen, the latter being aided by some, we trust not all, of the members of the board of examiners of the several States. There has been from the very inception of these boards a feeling of antagonism between them and the faculties, and the reason for this has been plain to the intelligent observer of events.

The laws formulated and adopted in the various States were based, we are willing to believe, upon an honest desire to improve the standing of the profession. While this object cannot, in the opinion of many, be attained by law, it is regarded by others as the best method to secure improvement. If the history of the world had been intelligently read, it would have been observed that statutes never made a higher moral or intellectual life, but simply remained a repressive force to reduce the power of evil. It is too late now to argue for the good or ill results of State laws governing dentistry. They are here and will, it is presumed, remain an active power in professional work. It is to be deplored that some members of these boards should seek to open, or rather continue, a controversy that had, in a measure, quieted down.

In the present number will be found a paper prepared by a member of the New Jersey Board. It is admitted to our pages upon the established rule of the journal to hear all sides of a question, although it does not seem to be written in the spirit of conciliation so necessary at this period of the controversy, indeed, it seems to go to the profession with all the force of a declaration of war made by the writer against the dental colleges of the United States.

It must be apparent, in reading this paper, that the self-laudatory spirit is not one of the attributes lacking in one member of the New Jersey State Board. A few sentences are quoted in illustration of this. "A *résumé* of the question in regard to the influence of examining boards on dental education would necessarily become a history of the advance of modern dentistry. . . . I think . . . the colleges are not working entirely from a philanthropic stand-point, *while the examining boards are*. . . . Any one who has been an unprejudiced observer . . . must admit that the boards have . . . been the means of providing for the *student a higher and better education*."

It may not be considered out of place to remark in reply to these quotations that dental colleges were never organized upon a philanthropic basis, nor is it generally understood that the motives which influence the members of State boards are wholly altruistic in character. The one represents an effort to educate to a higher standard with all the means possible at command, recognizing at the same time that the work is a business, and must be conducted according to business principles or failure will result. As a certain portion of good inheres in all business relations, so in this, but it must not be permitted to overmaster the law of self-preservation. The evidences of philanthropic virtue in State boards has not as yet assumed obtrusive proportions, but the profession can patiently wait for the evidence that should be forthcoming as the controversy extends. It is in evidence that the members of these organizations are not paid more than their expenses and frequently not even that, but they have what is dearer to a certain class of men, power.

All members of these boards are not thus influenced; indeed, we know that the good of the profession is the sole motive which actuates many, perhaps a majority, but whether it is with all may be seriously questioned.

The following advice, quoted from Dr. Crouse, would be excellent if it were at all new, but as it is not we repeat it here simply to answer an unjust criticism. "Now as to the student's mechanical adaptability, . . . if he hasn't it in his fingers . . . he will never make a dentist, and of this fact the colleges have, as yet, taken no notice. . . . Therefore, the first six weeks of the college course should be spent in finding out who are properly qualified by nature, as well as by training, to be dentists."

The ignorance of college work displayed in this quotation is most remarkable. It is hardly to be questioned but that every effort has been made for years, during the first session or Freshman year, to examine critically into the inclination and ability of each separate student. It is a difficult and not always a pleasant task, but yet a duty each Dean must perform. In this effort to weed out the incapables, we have no doubt but that each Dean has discovered a decided opposition from the individual as well as parents and guardians. It is rarely that good advice of this character will be received in the spirit in which it is given. To expel a man because his abilities are supposed not to be of the highest order has not yet become a law of colleges, and we hope it may never be so long as the possibilities of development are without limitations.

We cannot follow this unfair and untruthful denunciation of college work. It would be waste of time to undertake to meet in detail charges based wholly on assumptions. No one can truthfully say of colleges as a whole that they are guilty of this or that wrong, or deserve commendation for this or that good accomplished. A very close and very intimate relation is needed for this, not obtainable even by those who are in daily connection with college work. Hence no member of one college would be found daring enough to assert any ill of a sister institution, but in proportion to the ignorance of the man will be his readiness to traduce the work.

The objection to State boards does not apply to these as servants of the State under a recognized law, but to the fact that the colleges have not confidence in them as at present constituted. It would seem not difficult to understand why men who devote their time and have exhausted their best abilities in instruction, have not much patience with men appointed as examiners by a governor through political favoritism. Ability to examine, as well as to teach, is acquired only by long years of experience, superadded to a thorough knowledge of the subject, and cannot be attained by a few hours study of "Questions and answers." It may be said that lack of knowledge must be exceptional with members of examining boards. We do not care to believe it true of the majority, but it is certainly of some, else why this query in substance and not singular in the experience of educators,—"Can't you give me a series of questions, I have just been appointed on the State board?" This kind of thing destroys confidence.

Our medical friends seem to be in no better shape. The medical board in Pennsylvania adopts the same tactics as the writer in the present number in quoting from examination papers, a proceeding not in good taste and an undeserved reflection on the schools generally.

A correspondent from Paris in one of our prominent medical journals thus ventilates his opinion of State boards. "Nine-tenths of the State Examining Boards in the United States could not answer one-tenth of the examination questions they ask. It is a source of amazement that the members of the true profession in any country, especially in the United States, should endure legislation that enchains them as to the bounds and limits in which to exhibit their professional skill. . . . A profession forced to defend itself by protection can only acknowledge its weakness." These are strong words, and while we do not endorse them entirely, there is something in the ideas enumerated worthy of reflection.

The remedy for all this unpleasant friction is to accept the laws as we have them until changed or repealed. See that graduates of the best schools are placed on the examining boards, and, as a precautionary measure, consult the records of the colleges as to the standing of candidates for appointment, but above all, take the selection of men out of the hands of the governors of the several States. This can only be done by legislative action, but it should be the first move in the direction of reform.

Honest criticism, based on absolute knowledge of college work, should be encouraged, but it must be conducted in a spirit of justice to all concerned. It should be remembered that colleges to be successful must be conducted upon a well-arranged system, and it is obligatory that this has for its foundation the instruction of the students, by any and all means attainable within honorable lines. In accomplishing this, if good can be done to a large class of patients it is well, and every right-thinking mind should rejoice, but it is not the purpose for which colleges were established, nor will it be possible to force them to consider it by any amount of ill-advised and open denunciation.

